# Nicotine Contamination in Particulate Matter Sampling

**DOI:** 10.3390/ijerph6020601

**Published:** 2009-02-09

**Authors:** Yueh-Hsiu Chiu, Jaime E. Hart, Thomas J. Smith, S. Katharine Hammond, Eric Garshick, Francine Laden

**Affiliations:** 1 Exposure, Epidemiology and Risk Program, Department of Environmental Health, Harvard School of Public Health, Boston, MA 02215, USA; 2 Channing Laboratory, Brigham and Women’s Hospital and Harvard Medical School, Boston, MA 02115, USA; 3 School of Public Health, University of California, Berkeley, CA 94720, USA; 4 Pulmonary and Critical Care Medicine Section, VA Boston Healthcare System, Boston, MA 02130, USA; 5 Department of Epidemiology, Harvard School of Public Health, Boston, MA 02115, USA

**Keywords:** Nicotine, particulate matter, sampling filter, contamination, secondhand smoke exposure, cigarette smoke

## Abstract

We have addressed potential contamination of PM_2.5_ filter samples by nicotine from cigarette smoke. We collected two nicotine samples – one nicotine sampling filter was placed inline after the collection of PM_2.5_ and the other stood alone. The overall correlation between the two nicotine filter levels was 0.99. The nicotine collected on the “stand-alone” filter was slightly greater than that on the “in-line” filter (mean difference = 1.10 μg/m^3^), but the difference was statistically significant only when PM_2.5_ was low (≤ 50 μg/m^3^). It is therefore important to account for personal and secondhand smoke exposure while assessing occupational and environmental PM.

## 1. Introduction

One common problem of environmental exposure sampling is the possibility of capturing substances other than target chemicals or particles. Of particular concern is contamination by cigarette smoking in occupational studies where particulate matter (PM) is being used as the marker of exposure to environmental and industrial sources. Potential problems are the possibility of adsorption of vapor phase cigarette constituents or the possibility of contamination of cigarette smoke particles resulting in an over-estimation of PM exposures determined using filters [1]. In this study, we addressed potential contamination by nicotine, a marker of cigarette smoking, in PM_2.5_ (particulate matter less than 2.5 microns in diameter) samples from US trucking industry drivers. The exposure of interest in this group is vehicle exhaust, primarily from diesel engines, but these individuals are allowed to smoke while driving, leading to possible over-estimation of their exposures to vehicle-related PM_2.5_.

## 2. Results and Discussion

### 2.1. Results

A total of 16 samples were collected from the truck cabs of smoking drivers. Table 1 shows the distributions of PM_2.5_, the “stand-alone” and “inline” nicotine concentrations, and the paired differences between the two measurements of nicotine concentrations, stratified by PM_2.5_ concentration.

The average “stand-alone” nicotine concentration was 8.20 (SD 7.29) μg/m^3^ and the average “inline” nicotine concentration was 7.10 (SD 7.03) μg/m^3^. PM_2.5_ was bimodally distributed around 50 μg/m^3^, while the median level was 34.6 μg/m^3^ for low PM_2.5_ group (≤ 50 μg/m^3^) and was 111.9 μg/m^3^ for high PM_2.5_ group (>50 μg/m^3^). In addition, nicotine levels were higher in samples with higher PM_2.5_ concentrations. There was an average absolute difference of 1.10 μg/m^3^ (*p*=0.0003) between the two measurements of nicotine concentrations, but the differences were statistically significant only in the low PM_2.5_ group (*p*<0.05).

**Figure 1.**
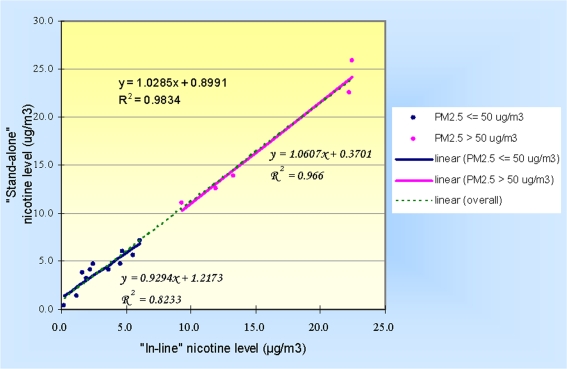
Correlation between “stand-alone” filter and “inline” filter nicotine concentrations.

The “stand-alone” versus “inline” nicotine concentrations are plotted in Figure 1. They were highly correlated and the slope of the regression line was close to 1, both overall and when stratified by PM_2.5_ concentration. The overall Pearson’s correlation coefficient was 0.99 (*p*<0.0001), and was 0.98 (*p*=0.0027) for the high PM_2.5_ group and 0.91 (*p*=0.0001) for the low PM_2.5_ group.

**Figure 2.**
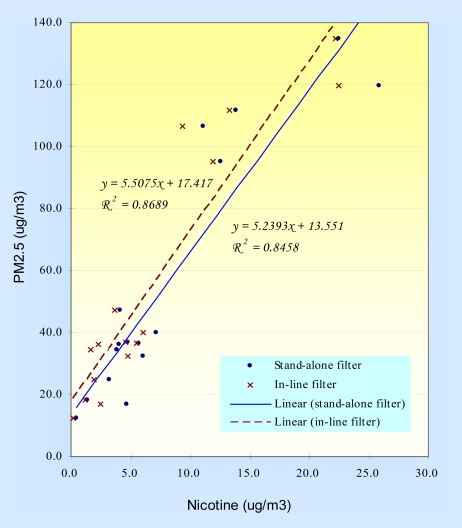
Correlations between PM_2.5_ concentrations and nicotine concentrations using two different sampling designs.

To further investigate the association between PM_2.5_ and nicotine concentrations, we also examined their correlation (Figure 2). We found a strong correlation between both the “stand-alone” and “inline” nicotine concentrations and PM_2.5_ (Pearson’s *r* = 0.92 and 0.93, respectively). The slopes of the two regression lines, “stand-alone” nicotine vs. PM_2.5_ concentration (slope=5.5) and “inline” nicotine vs. PM_2.5_ concentration (slope=5.2), were similar, suggesting that the adsorption of nicotine to the PM_2.5_ filter was fairly systemic.

### 2.2. Discussion

Our study showed that the overall difference in nicotine concentrations measured between “stand-alone” filters and filters behind PM_2.5_ filters (“inline” filters) was small, indicating that PM_2.5_ samples are not largely contaminated by nicotine from cigarette smoke. In other words, “inline” nicotine concentrations are not largely underestimated (average value of 1.10 ±0.96 μg/m^3^). In support of this is the finding of two separate regression lines from the two nicotine measurements versus PM_2.5_, indicating that there is a small but systematic difference between them. Although firm conclusions are limited by sample size, it is possible that the underestimation of “inline” nicotine concentrations was significant only where PM_2.5_ concentrations were lower because the relative contribution of nicotine to overall mass is greater. The average percent of difference (calculated from dividing the absolute difference of the two nicotine levels by the “stand-alone” nicotine level and then times 100%) was larger in the low PM_2.5_ group, compared to the high group, suggesting more variability or larger measurement errors in the former group. Another explanation is that the PM_2.5_ sampling filter might have a small and fixed adsorption capacity for nicotine, which is independent of the particle loading – it appears as an offset of the regression line. This small amount of nicotine may only be detectable when the particle loading is also small. Also, the sample size was smaller in the group with higher PM_2.5_ levels.

We did not separately measure nicotine in the vapor and particulate phase of cigarette smoke, and it is expected based on the known distribution of nicotine in cigarette smoke that nicotine is present predominantly in the vapor phase [2]. We also attempted to estimate the total particles from cigarette smoke from the “stand-alone” nicotine levels, using the ratio 8.6 suggested by a previous study of railroad workers and experiments conducted in a well-mixed chamber [3]. However, when applying this ratio to our samples, 56% of the estimated cigarette smoke particles were greater than the mass on the PM_2.5_ filter. This might be due to the unique exposure scenario inside the truck cab. In addition, the constitutions in cigarette smoke may have changed over time. Further studies are needed to evaluate the potential contamination by particles from cigarette smoke on the PM_2.5_ samples.

The results from our study showed that samples with higher PM_2.5_ levels are associated with higher nicotine levels (Figure 2), suggesting that a large proportion of PM_2.5_ in a truck cab was derived from cigarette smoke. This implies that smoking in trucks or cars may relate to a potentially higher health risk, especially for transportation professionals who spend more time in these settings. In addition, although the contribution of nicotine to PM_2.5_ filters in our samples was relatively small, the contribution was proportionally greater at lower PM_2.5_ values. Our results indicate that it is important to consider the impact of secondhand and active smoking when sampling occupational and environmental exposures to airborne pollutants. Design methods include restricting exposure sampling to non-smokers or stratifying by cigarette smoke and secondhand smoke exposure [4,5]. Statistical modeling can also be used to control for cigarette smoke [6,7]. One problem with restriction and statistical modeling is that they only account for personal smoking but they might not adequately account for secondhand smoke exposures. Our study suggests that adjusting for nicotine as in our study might provide a way to adjust for PM filter contamination of nicotine related to cigarette smoking by deriving a correction factor – either overall or specific for different PM_2.5_ levels (see Table 1). Finally, other exposure metrics which are not largely affected by smoking, e.g. elemental carbon (EC) [8], are often used to assess workers’ occupational exposures to diesel airborne pollutants [9–13]. In conclusion, we suggest that it is important to account for personal and secondhand smoke exposure while assessing low level occupational and environmental exposures to particles, especially when PM_2.5_ level is less than 50 μg/m^3^. The collection of nicotine in this manner may be useful for adjusting PM_2.5_ levels for the impact of nicotine from cigarette smoke.

## 3. Materials and Methods

### 3.1. Sampling Location

PM_2.5_ and nicotine were measured in truck cabs of drivers who smoked. The participants were volunteers in the exposure assessment arm of the Trucking Industry Particle Study, a five-year national study of diesel exhaust exposure and lung cancer risk in four large unionized trucking companies [12].

### 3.2. Sampling Schemes

**Figure 3.**
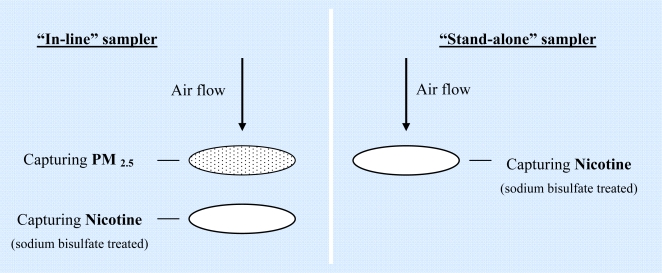
Sampling designs of “stand-alone” sampler and “inline” sampler.

The overall design of the exposure assessment and the methods used are described in detail elsewhere [2,12]. In brief, using an active sampler, PM_2.5_ was collected across a work shift on pre-weighed Teflon filters (Pall Co., Ann Arbor MI) after the particles larger than 2.5 μm were removed by a cyclone separator. We placed a 37mm Teflon-coated glass fiber filter (Emfab TX40HI20WW, Pallflex Corp., Putnam, CT) treated with sodium bisulfate to collect vapor phase nicotine behind the PM_2.5_ filter. To determine whether the second filter (i.e. the “inline sampler”) collecting vapor phase nicotine was underestimated as a result of adsorption of vapor phase nicotine on the first filter (collecting PM_2.5_), we measured nicotine levels with a “stand-alone” sodium bisulfate treated filter placed in parallel with the first sampler (Figure 3). Since nicotine is mainly present in the vapor phase, we used the nicotine concentration measured from the “stand-alone” filter as an estimate of total nicotine level from cigarette smoke [2]. To avoid interfering with the driver’s activities, the sampling system was mounted to the dashboard next to the driver. The nicotine filters were analyzed by desorption of the nicotine and quantification by gas chromatography with nitrogen selective detection [2]. PM_2.5_ filters were pre-weighed after humidity equilibrium (between 20–23 °C and between 35–45% relative humidity for at least 48 hours) in a chamber by an analytic balance (Micro-Gravimetric M5, Mettler Instruments Corp, Hightstown, NJ). After sampling, the PM_2.5_ filters were reweighed after humidity equilibrium to determine the weight gain [12]. Concentrations were calculated by dividing mass collected (μg) from the estimated volume of air sampled (m^3^).

### 3.3. Statistical Analysis

To quantify vapor phase nicotine that became adsorbed to the PM_2.5_ filter, we examined the difference of the “inline” and “stand-alone” nicotine concentrations using paired-t tests. We also assessed the correlations between PM_2.5_ levels and nicotine levels. All data analyses were performed in SAS (V9.1.3, SAS Institute Inc., Cary, NC). The protocol was approved by the Human Subjects Committees at the Harvard School of Public Health, Brigham and Women’s Hospital, and VA Boston Healthcare System.

## Figures and Tables

**Table 1.** Distributions of PM_2.5_ (μg/m^3^), “stand-alone” and “inline” nicotine concentration (μg/m^3^), absolute nicotine concentration difference (μg/m^3^).

		**PM2.5conc.**			**“Stand-alone” nicotine conc.**			**“In-line” nicotine conc.**			**Nicotine conc. difference [(“Stand-alone”) – (“In-line”)] a**			
		
	**N**	Mean	SD	Range	Mean	SD	Range	Mean	SD	Range	Mean	SD	Range	
**Overall**	16	56.5	41.5	(12.5–134.9)	8.20	7.29	(0.43–25.84)	7.10	7.03	(0.20–22.47)	1.10	0.96	(0.12–3.37)	*
**PM_2.5_ conc. b**														
*> 50 μg/m^3^*	5	113.7	14.8	(95.3–134.9)	17.2	6.58	(11.1–25.84)	15.8	6.10	(9.33–22.47)	1.33	1.27	(0.33–3.37)	
*≤50 μg/m^3^*	11	30.5	10.9	(12.5–47.1)	4.12	1.96	(0.43–7.16)	3.13	1.91	(0.20–6.06)	0.99	0.83	(0.12–2.25)	*

a Paired T-test was used to examine the absolute difference between “stand-alone” and “inline” nicotine concentrations. (* *p*-vale < 0.05)

b PM_2.5_ was bimodaly distributed around 50 μg/m^3^, which was chosen as the cut point. Subjects with nicotine conc. > 10 μg/m^3^ were all exposed to PM_2.5_ conc. > 50 μg/m^3^; subjects with nicotine conc. ≤10 μg/m^3^ were all exposed to PM_2.5_ conc. ≤ 50 μg/m^3^.
